# Interactive dose shaping part 2: proof of concept study for six prostate patients

**DOI:** 10.1088/0031-9155/61/6/2471

**Published:** 2016-03-07

**Authors:** Cornelis Ph Kamerling, Peter Ziegenhein, Florian Sterzing, Uwe Oelfke

**Affiliations:** 1 Joint Department of Physics, The Institute of Cancer Research and The Royal Marsden NHS Foundation Trust, London, SM2 5NG, UK; 2 Department of Medical Physics in Radiation Oncology, German Cancer Research Center (DKFZ), Im Neuenheimer Feld 280, 69120 Heidelberg, Germany; corijn.kamerling@icr.ac.uk; uwe.oelfke@icr.ac.uk

**Keywords:** interactive treatment planning, radiation therapy, treatment planning software, graphical user interface

## Abstract

Recently we introduced interactive dose shaping (IDS) as a new IMRT planning strategy. This planning concept is based on a hierarchical sequence of local dose modification and recovery operations. The purpose of this work is to provide a feasibility study for the IDS planning strategy based on a small set of six prostate patients.

The IDS planning paradigm aims to perform interactive local dose adaptations of an IMRT plan without compromising already established valuable dose features in real-time. Various IDS tools were developed in our in-house treatment planning software Dynaplan and were utilized to create IMRT treatment plans for six patients with an adeno-carcinoma of the prostate. The sequenced IDS treatment plans were compared to conventionally optimized clinically approved plans (9 beams, co-planar). For each patient, several IDS plans were created, with different trade-offs between organ sparing and target coverage. The reference dose distributions were imported into Dynaplan. For each patient, the IDS treatment plan with a similar or better trade-off between target coverage and OAR sparing was selected for plan evaluation, guided by a physician.

For this initial study we were able to generate treatment plans for prostate geometries in 15–45 min. Individual local dose adaptations could be performed in less than one second. The average differences compared to the reference plans were for the mean dose: 0.0 Gy (boost) and 1.2 Gy (PTV), for ${{D}_{98\%}}:-1.1$ Gy and for ${{D}_{2\%}}:1.1$ Gy (both target volumes). The dose-volume quality indicators were well below the Quantec constraints. However, we also observed limitations of our currently implemented approach. Most prominent was an increase of the non-tumor integral dose by 16.4% on average, demonstrating that further developments of our planning strategy are required.

## 1. Introduction

Intensity modulated radiation therapy (IMRT) is known to provide a high degree of control over clinical dose distributions in high precision radiation therapy. A high spatial conformity can be established for the prescribed dose to the tumor volume while dose in adjacent healthy tissue can be minimized. Treatment planning encompasses finding a clinically optimal set of fluence intensities. This search is usually performed as solving an inverse planning problem (Bortfeld 2006), which typically relies on the iterative optimization of a piece-wise quadratic cost function. This function includes pre-segmented volumes of interest and dose constraints which are weighted by penalty factors. The optimization problem is solved by a treatment planning system (TPS), which generates a 3D dose distribution as function of the fluence amplitudes of the radiation field. Subsequently, 3D and 2D dose displays, dose volume histograms and other quality indicators have to be evaluated by the therapist. Based on this plan assessment, the parameters of the cost function have to be revised manually.

The indirect approach of finding the clinically optimal dose distribution suffers from various inherent shortcomings:

(i) The control of local dose features is limited to segmented volumes of interest. This makes it for example difficult to remove cold or hot spots.
(ii) There is no intuitive mapping between the parameters of the objective function and the resulting dose distribution. Generating a treatment plan may require a tedious loop of manual constraint adaptation and re-optimization, yielding to a substantial workload for the therapist.
(iii) If the patient geometry changes between fractions, it is often difficult to adjust the initial treatment plan accordingly. In the worst-case the whole optimization process has to be repeated. This involves the re-calculation of dose-influence data or plan databases (depending on the optimization method), and a repetition of the manual parameter tuning process.

To overcome these shortcomings a new planning paradigm was proposed: interactive dose shaping (IDS) (Ziegenhein *et al* 2016). In contrast to conventional treatment planning, IDS is performed by direct interaction with a 3D dose distribution through a graphical user interface (GUI). This interactive work-flow allows the therapist to directly utilize his clinical knowledge of the impact of a 3D dose distribution for a given patient geometry and diagnosis, by modifying the dose directly and locally. This approach requires that dose shaping can be performed in real-time, e.g. the therapist does not have to wait longer than one second before he can inspect the result of his actions.

The development of IDS can be categorized in two problem domains:

(i) The development, implementation and evaluation of intelligent dose modification. The key operation that facilitates dose shaping is a two-step modification and recovery strategy, which heavily relies on a real-time dose calculation algorithm (Ziegenhein *et al* 2013b).
(ii) The development, implementation and evaluation of a novel treatment planning software, comprising of a set of interactive 3D dose manipulation tools which enable intuitive local changes of a dose distribution in real-time. This requires an intuitive, responsive 3D GUI and speed-optimised algorithms with minimal latency (Kamerling *et al* 2013).

In this work we present a proof of concept study for IDS. The IDS software was utilized to create a treatment plan for six clinical patients with an adeno-carcinoma of the prostate. The obtained IDS fluence maps were sequenced and then compared with conventionally optimized clinically approved plans.

## 2. Materials and methods

### 2.1. Interactive dose shaping

**Figure 1.**
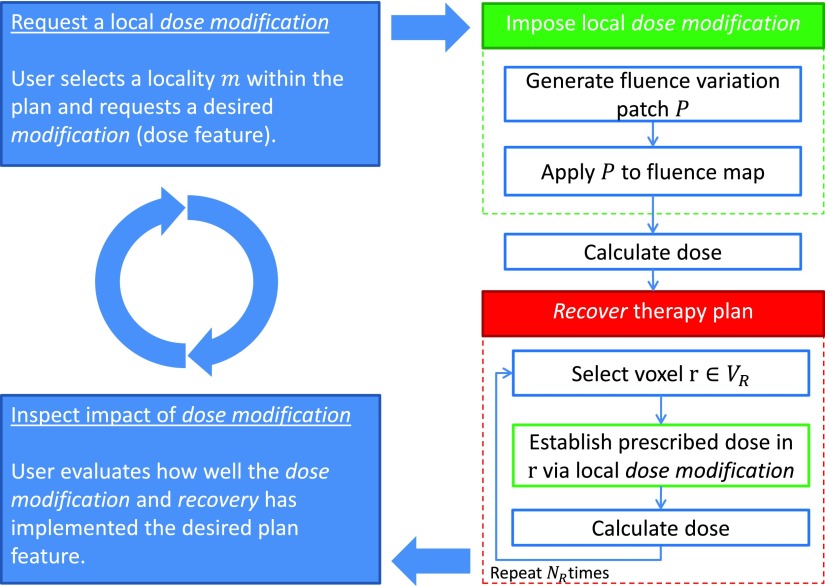
Two-step dose modification and recovery algorithm. The blue boxes indicate user actions: requesting a modification and inspecting its impact. The green box represents the implementation of the local dose modification. The red box illustrates the recovery process.

The key operation driving IDS is the two-step dose modification and recovery (DMR) strategy, which is summarized in figure 1. This algorithm is initiated by a request for a local dose modification (top right blue box), either by a user through a GUI or by an algorithm. The first step, a dose modification (top right green box), is a direct change of dose in a voxel. This change is obtained by adjusting the current fluence map by applying a fluence patch *P*, which enforces the requested dose difference. The fluence adaption will naturally lead to unintended and unwanted dose deviations outside of the selected, local area of modified dose. The subsequent recovery step (bottom right red box) identifies these voxels $r\in {{V}_{R}}$ and aims to recover their original dose by imposing a set of dose modifications (as in the first step). The number of voxels which are recovered for a single instance of DMR is determined by *N*_*R*_. Details about the strategy and algorithmic implementation of the DMR steps are provided in Ziegenhein *et al* (2016).

The computational bottleneck is the 3D dose calculation, which has to be performed for each modification. The computation time of a single DMR instance is, therefore, directly proportional to *N*_*R*_. The real-time requirement limits the number of voxels which can be recovered for a single DMR instance. The IDS dose calculation engine is based on Bortfeld *et al* (1993) and was optimized in terms of locality, speed and latency. The algorithm exploits modern multi-core central processing unit (CPU) technology and is tightly coupled to both the DMR and GUI engine. On a modern low-cost Intel i7 desktop computer, *N*_*R*_ can be set to 15 while the computation time stays below one second.

### 2.2. Dynaplan

**Figure 2.**
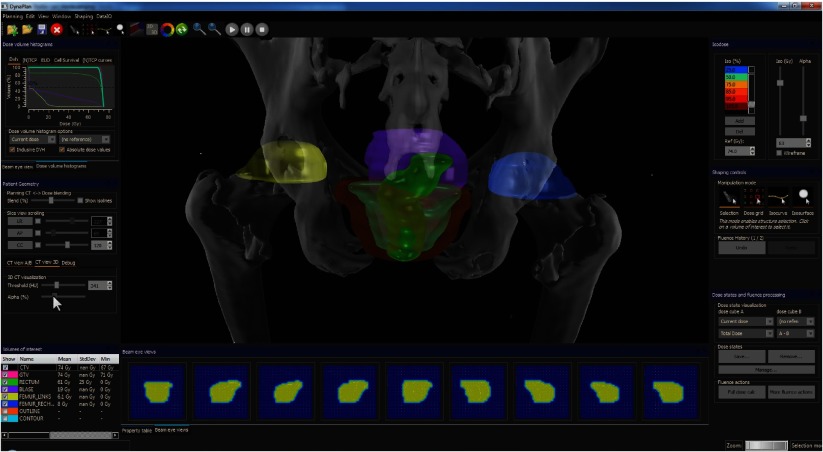
Screenshot from Dynaplan, showing a 3D visualization of a prostate patient, fluence maps, quality indicators and various other widgets.

Dynaplan (figure 2) is a software prototype, consisting of a 3D GUI for dose manipulation and visualization, including a 2D and 3D interface which hosts a slice view, volume of interest (VOI) visualization, beam’s eye view, volume rendering, quality indicators and graph plotting. To guarantee responsiveness, all algorithms were optimized for speed and low latency by utilizing various degrees of parallelization. 3D graphics were based on the extension of the 3D graphics engine Ogre3D^3^3Ogre3D is an open-source multi-platform 3D graphics engine which abstracts over OpenGL (Khronos Group, Beaverton, OR, USA) and Direct3D (Microsoft, Redmond, WA, USA) (www.ogre3d.org).. All windows, dialogues and other widgets were implemented using Qt^4^4Qt is a cross-platform application and GUI framework by Digia (Helsinki, Finland) (www.qt-project.org)., 2D plots using Qwt^5^5Qwt is an open-source GUI library (mainly focussing on 2D plotting) based on Qt (qwt.sourceforge.net).. The computation intensive algorithms run in parallel through OpenMP (Dagum and Menon 1998) and AVX^6^6Advanced Vector Extensions (AVX) is a set of instructions for performing single instruction multiple data (SIMD) computations on modern CPUs.. For some algorithms, for which latency is of little importance, graphical processing unit (GPU) implementations were realized. To enable convenient user navigation and manipulation simultaneously, the SpaceNavigator^7^7SpaceNavigator is a product from 3Dconnexion, Waltham, MA, USA (www.3dconnexion.com). 3D mouse is supported.

### 2.3. IDS tools

The DMR algorithm can be used as building block for various IDS tools. Three of these tools were implemented into Dynaplan. The most straightforward IDS tool is single voxel manipulation. With this tool, the therapist can select a voxel in a 3D dose grid and subsequently click a button to request a local dose increase or decrease. This results in the computation of a single DMR instance. A second tool allows for isodose curve manipuation, which allows a user to drag isodose lines using a computer mouse. The curve change is translated in a set of DMR instances which are executed consecutively. The third tool offered by Dynaplan is the isodose surface manipulation tool, which enables dose shaping by intuitively clicking on an isodose surface. To guide the user interaction, the position of the modification is indicated by a sphere while the mouse cursor hovers the isodose surface. The sphere’s radius controls the locality of the modification, which is implemented by the DMR algorithm. Screenshots of the isodose surface manipulation tool as used in this work can be found in Kamerling *et al* (2013).

### 2.4. Proof of concept study

To evaluate the clinical potential of Dynaplan, a planning study was performed for a collective of six patients, each diagnosed with adeno-carcinoma of the prostate and treated with step-and-shoot IMRT at the German Cancer Research Center. The patient geometry and beam setup were defined in Virtuos (Bendl *et al* 1995). All plans consisted of a co-planar nine beam configuration. The treatment plans obtained by IDS were compared to the clinically approved reference plans. These plans were generated by conventional IMRT optimization, utilizing the Konrad software (Preiser *et al* 1998) and a standard IMRT leaf sequencing algorithm provided by Siemens (Munich, Germany). For all reference plans, the 3D dose distributions and dose prescription were available. Pencil beam dose calculation was performed on a grid with a voxel size of $1.95\times 1.95\times 2.0$ mm^3^. The number of voxels per transversal slice was $256\times 256$. The number of slices, which varied from patient to patient, ranged from 154 to 234.

#### 2.4.1. Segmented volumes and dose prescription.

**Figure 3.**
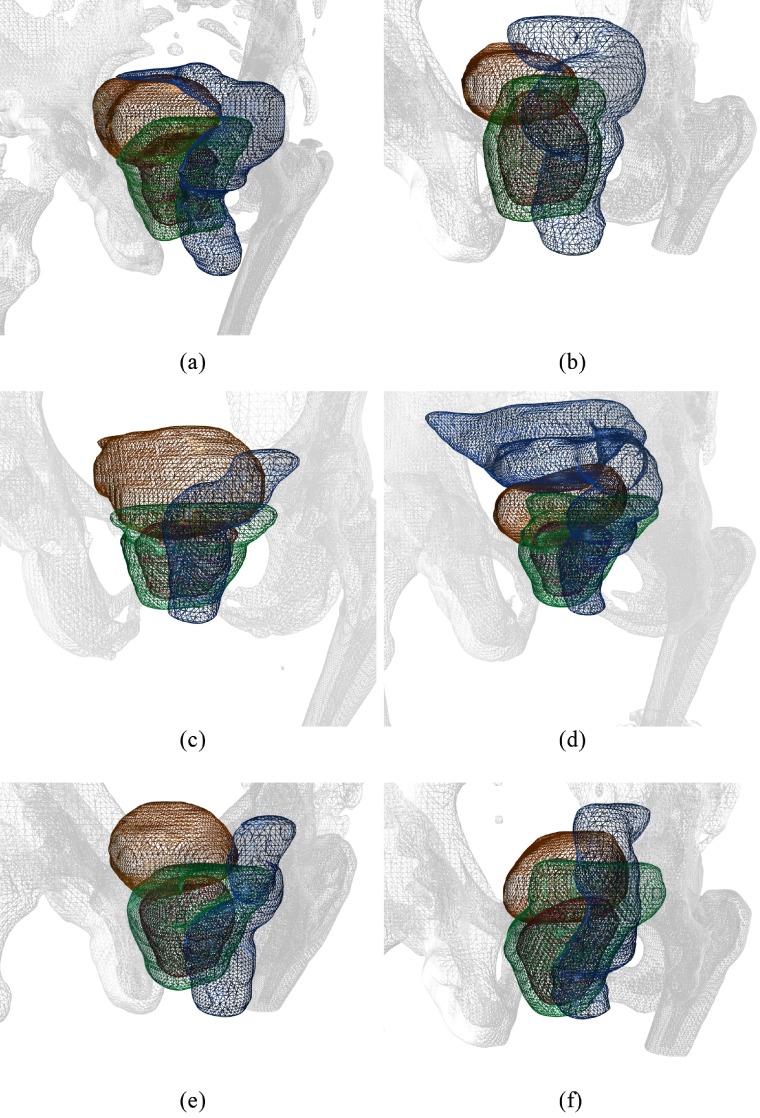
The six patient geometries for the prostate treatment planning study. Colored triangle meshes are shown for the PTV (green), boost volume (red), rectum (blue) and bladder (orange). A bone segmentation of the planning CT is shown as grey triangle mesh. (a) Patient 1. (b) Patient 2. (c) Patient 3. (d) Patient 4. (e) Patient 5. (f) Patient 6.

Figure 3 depicts the patient setup for each of the six patients. The orientation can be deduced from the triangulated bone segmentation (in grey). The individual structures are shown as colored triangle mesh: the planning target volume (PTV) in green, and rectum and bladder in blue respectively orange as organs at risk (OARs). Moreover, the planning protocol at the German Cancer Research Center required the definition of a simultaneous integrated boost volume (in red), which had a higher dose prescription than the PTV. The dose prescriptions ranged from 73 to 76 Gy for the boost volume and 70 to 73 Gy for the PTV.

#### 2.4.2. IDS treatment plans.

The patient geometry and beam setup were imported into Dynaplan. Treatment planning was performed by one of the software developers, without prior knowledge of the reference dose distribution. The treatment goals were pursued in a hierarchical order. First, an initial dose distribution was established by open fields such that ${{D}_{50\%}}$ of the PTV satisfied the average dose prescription of the PTV and boost volume. Then, the isodose surface manipulation tool was used by manual GUI interaction to shape the dose distribution, so that the rectum and bladder OAR were spared. The initial goal for OAR sparing was satisfying each Quantec dose-volume constraint *V*_*x*_. For the rectum: ${{V}_{50}}&lt;50\%$, ${{V}_{60}}&lt;35\%$, ${{V}_{65}}&lt;25\%$, ${{V}_{70}}&lt;20\%$, ${{V}_{75}}&lt;15\%$ (Michalski *et al* 2010), and for the bladder: ${{V}_{65}}&lt;50\%$, ${{V}_{70}}&lt;35\%$, ${{V}_{75}}&lt;25\%$, ${{V}_{75}}&lt;15\%$ (Viswanathan *et al* 2010), with *x* in Gy. After reaching these goals, further OAR sparing was explored as trade-off with target coverage. Satisfying treatment plans were saved as dose state. For each patient, the planning time was recorded. IDS treatment planning was performed on an eight-core Intel (Santa Clara, CA, USA) Xeon workstation PC, equipped with an Nvidia (Santa Clara, CA, USA) Quadro K5000 GPU and 3Dconexxion (Boston, MA, USA) 3D mouse.

#### 2.4.3. Plan evaluation.

Per patient, several IDS plans were created before the dose distribution was imported into Dynaplan. The IDS treatment plan with a similar or better trade-off between target coverage and OAR sparing was selected for plan evaluation, guided by a physician. To generate a deliverable treatment plan, the fluence maps were sequenced by the Siemens sequencer, for a multi-leaf collimator (MLC) with 5 mm leafs and a stratification with 7 equidistant fluence levels. The same MLC was utilized for the reference plans. The number of segments (leaf-configurations) for both the IDS plans and the reference plans were reported. No plan normalization was applied.

Several quality indicators were calculated for plan evaluation and comparison. For the boost volume and PTV, mean and median dose were calculated, as well as ${{D}_{98\%}}$ and ${{D}_{2\%}}$. A dose volume histogram (DVH) was plotted for each patient.

A conformity index *CI* was computed as in van’t Riet *et al* (1997) using a dose threshold of 95% from the prescribed dose:

1 \begin{eqnarray*}CI=C{{I}_{1}}\centerdot C{{I}_{2}}=\frac{{{V}_{\text{t},\text{ref}}}}{{{V}_{\text{t}}}}\centerdot \frac{{{V}_{\text{t},\text{ref}}}}{{{V}_{\text{ref}}}}\end{eqnarray*}

where ${{V}_{\text{t},\text{ref}}}$ is the volume of the target receiving at least the prescribed dose, *V*_t_ is the target volume, and *V*_ref_ is the whole patient volume receiving at least the prescribed dose. $\frac{{{V}_{\text{t},\text{ref}}}}{{{V}_{\text{t}}}}$ expresses the fraction of the target volume at least receiving the prescribed dose and is referred to as *CI*_1_. $\frac{{{V}_{\text{t},\text{ref}}}}{{{V}_{\text{res}}}}$ indicates how much high dose (higher than the prescribed dose) is delivered adjacent to the target volume, i.e. how tight the dose gradient surrounds the target volume. This term is referred to as *CI*_2_.

A homogeneity index (*HI*) was expressed a standard deviation of the dose distribution. For the rectum and bladder OAR, the dose-volume quality indicators specified by Quantec were determined.

The non-tumor integral dose *NTID* for a VOI *j* was calculated based on D’Souza (2003):

2 \begin{eqnarray*}NTI{{D}_{j}}={{\rho}_{j}}\frac{{{V}_{j}}}{{{N}_{j}}}\underset{i}{\overset{{{N}_{j}}}{\sum}}\,{{D}_{i}},\end{eqnarray*}

where *V*_*j*_ is the volume, *N*_*j*_ is the number of voxels *i* and *D*_*i*_ is the dose deposited in voxel *i*. For this work, the non-tumour volume of interest was derived by subtracting the PTV from the body VOI. A constant value of one was assumed for ${{\rho}_{j}}$.

## 3. Results

The results of the prostate patient planning study are listed in tables 1–4. For each patient, quality indicator values for both the IDS treatment plan and the reference plan are listed. The last row of each table indicates the mean difference (indicated by *mean diff*) between the IDS plan and the reference plan for the respective quality indicator. Median is abbreviated as *med*, integral dose as *NTID*. The PTV quality indicators are computed for the whole PTV volume, i.e. including the boost region.

**Table 1.** Quality indicators for boost volume.

Plan	Mean (Gy)	Med (Gy)	*CI*_1_ (1)	*CI*_2_ (1)	*D*_98_ (Gy)	*D*_2_ (Gy)	*HI* (Gy)
Patient 1 IDS	76.0	76.3	0.95	0.46	71.3	79.6	2.1
Patient 1 reference	76.0	76.1	0.98	0.68	72.1	78.5	1.4
Patient 2 IDS	73.0	72.8	0.98	0.56	69.6	75.3	1.5
Patient 2 reference	73.0	73.6	0.98	0.72	69.7	75.1	1.4
Patient 3 IDS	76.0	75.7	0.97	0.42	72.0	80.2	2.8
Patient 3 reference	76.0	75.9	0.98	0.71	72.6	79.1	2.2
Patient 4 IDS	76.0	75.8	0.96	0.30	70.8	79.9	2.6
Patient 4 reference	76.0	76.1	0.97	0.73	72.1	79.4	2.3
Patient 5 IDS	74.0	74.0	0.97	0.46	69.7	78.3	2.0
Patient 5 reference	74.0	74.1	1.00	0.70	72.0	76.4	1.2
Patient 6 IDS	74.0	73.8	0.97	0.51	69.7	77.0	2.3
Patient 6 reference	74.0	74.4	0.99	0.78	70.6	76.0	1.9
Mean diff	0.0	−0.3	−0.02	−0.27	−1.0	1.0	0.5

**Table 2.** Quality indicators for PTV.

Plan	Mean (Gy)	Med (Gy)	*CI*_1_ (1)	*CI*_2_ (1)	*D*_98_ (Gy)	*D*_2_ (Gy)	*HI* (Gy)
Patient 1 IDS	73.0	73.8	0.93	0.68	62.2	78.8	4.0
Patient 1 reference	72.5	72.1	1.00	0.90	64.9	77.7	3.4
Patient 2 IDS	70.7	71.2	0.90	0.79	62.3	75.3	3.2
Patient 2 reference	70.5	70.5	0.92	0.90	65.1	75.1	2.8
Patient 3 IDS	73.4	73.8	0.97	0.61	65.6	79.3	4.8
Patient 3 reference	72.2	71.8	0.95	0.90	65.4	78.3	5.1
Patient 4 IDS	75.2	75.8	0.97	0.52	64.9	79.9	4.9
Patient 4 reference	72.8	72.9	0.97	0.80	65.6	78.6	4.9
Patient 5 IDS	72.4	72.3	0.96	0.70	65.4	77.4	2.9
Patient 5 reference	71.0	71.7	0.97	0.90	66.0	75.6	2.7
Patient 6 IDS	71.5	72.1	0.96	0.61	61.5	77.0	5.3
Patient 6 reference	70.2	70.6	0.94	0.96	61.3	76.0	6.0
Mean diff	1.2	1.6	−0.01	−0.24	−1.1	1.1	0.0

**Table 3.** Quality indicators for rectum.

Plan	*V*_50_ (%)	*V*_60_ (%)	*V*_65_ (%)	*V*_70_ (%)	*V*_75_ (%)
Patient 1 IDS	19.6	11.0	7.0	3.3	0.5
Patient 1 reference	35.8	9.8	6.2	3.0	0.1
Patient 2 IDS	11.4	4.6	1.9	0.1	0.0
Patient 2 reference	15.3	9.5	5.5	0.7	0.0
Patient 3 IDS	7.1	3.9	2.7	1.1	0.2
Patient 3 reference	5.8	2.8	1.7	0.8	0.0
Patient 4 IDS	27.8	15.9	10.1	4.6	0.8
Patient 4 reference	25.8	13.9	8.7	3.9	0.0
Patient 5 IDS	22.7	9.5	4.4	1.0	0.0
Patient 5 reference	23.7	12.3	7.6	1.7	0.0
Patient 6 IDS	31.7	18.8	12.9	6.3	0.6
Patient 6 reference	30.8	19.6	14.2	8.3	0.0
Mean diff	−2.8	−0.7	−0.8	−0.3	0.3

**Table 4.** Quality indicators for bladder and body contour.

Plan	*V*_65_ (%)	*V*_70_ (%)	*V*_75_ (%)	*V*_80_ (%)	*NTID* (l Gy)
Patient 1 IDS	11.7	4.8	0.4	0.0	141
Patient 1 reference	12.3	7.4	0.6	0.0	119
Patient 2 IDS	16.8	8.4	0.0	0.0	132
Patient 2 reference	20.4	8.0	0.3	0.0	113
Patient 3 IDS	8.5	3.6	0.7	0.0	112
Patient 3 reference	10.1	4.2	0.6	0.0	106
Patient 4 IDS	10.2	6.9	2.3	0.0	114
Patient 4 reference	8.1	5.1	0.5	0.0	91
Patient 5 IDS	10.4	7.1	2.4	0.0	102
Patient 5 reference	13.6	4.7	0.0	0.0	94
Patient 6 IDS	10.6	5.6	0.8	0.0	131
Patient 6 reference	7.7	4.7	1.1	0.0	106
Mean diff	−0.7	0.4	0.6	0.0	17.2

The quality indicators for the boost volumes are listed in table 1. The mean dose was equal to the prescribed dose for all IDS and reference plans. The median dose was slightly lower for the IDS plans. *CI*_1_ expresses the fraction of the target volume, at least receiving 95% of the prescribed dose. This term was equal or slightly lower for the IDS plans. *CI*_2_ indicates how much high dose (higher than 95% of the prescribed dose) is delivered adjacent to the target volume. A lower value means that more high dose is spilled around the tumor volume. This term was considerably lower for the IDS plans. *D*_98_ was slightly smaller, *D*_2_ slightly larger for IDS. *HI* was larger.

The quality indicators for the PTVs are listed in table 2. Both the mean and median dose were consistently larger, ranging from a 0.3% to 4.0% increase compared to the reference plans. *CI*_1_ deviated on average only 1%. *CI*_2_ was considerably lower for the IDS plans. *D*_98_ was sometimes larger, sometimes smaller for the IDS plans. *D*_2_ was slightly larger. The mean differences for both *D*_98_ and *D*_2_ were very similar to the values of the boost volume. The *HI* was sometimes larger for the IDS plans, sometimes for the reference plans. The mean deviation is zero.

The quality indicators for the rectum are listed in table 3. All Quantec constraints were met. *V*_50_ was on average lower for the IDS treatment plans, with a very pronounced difference for patient 1. For *V*_60_, *V*_65_ an *V*_70_, there was no clear advantage for one of the methods. On average, the values for IDS were a bit better. *V*_75_ was close to zero, for all reference plans. The IDS values were a bit larger, but did not exceed 0.8%.

The quality indicators for the bladder are listed in table 4. All Quantec constraints were met for both the IDS and reference plans. For *V*_65_, *V*_70_, *V*_75_, the maximum volume deviation was 3.6%. The mean difference for *V*_65_ was slightly better for the IDS plans. *V*_70_ and *V*_75_ were on average marginally better for the reference plans. *V*_80_ was zero for all plans. Table 4 also presents *NTID* for the body contour. *NTID* for the IDS plans was higher than for the reference plans, with a mean difference of 17.2 l Gy corresponding to 16.4%.

**Figure 4.**
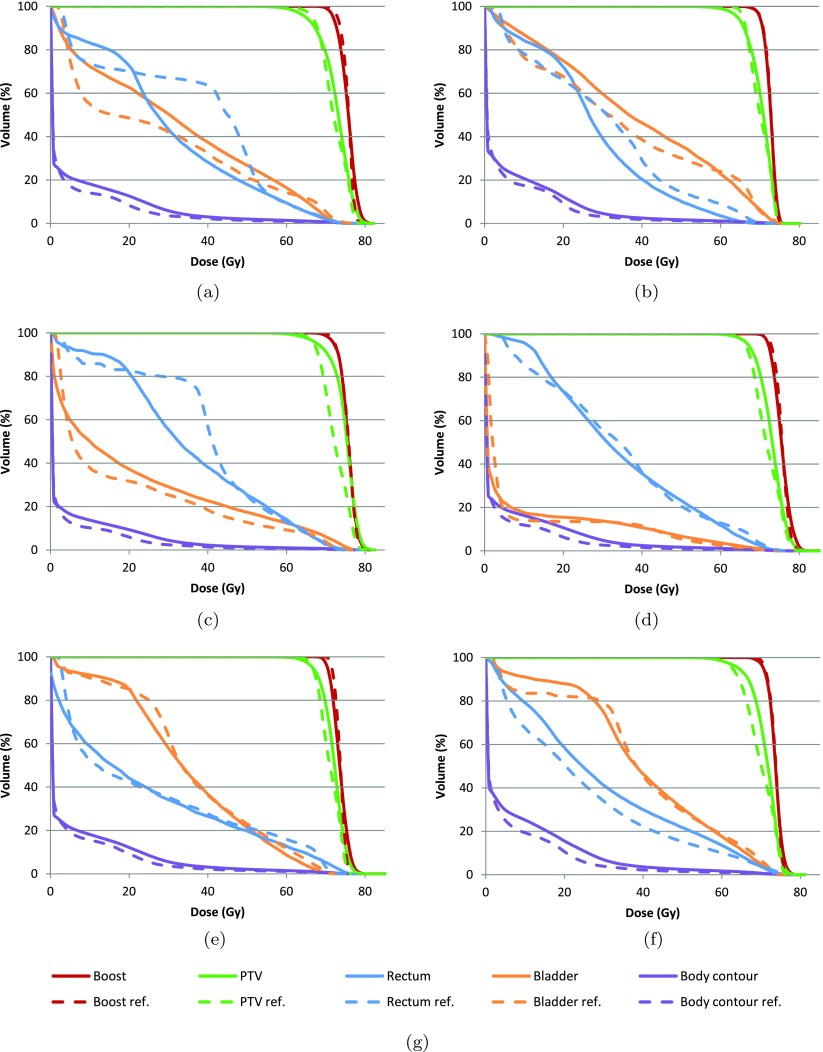
DVHs for each patient of the prostate case study. (a) Patient 1. (b) Patient 2. (c) Patient 3. (d) Patient 4. (e) Patient 5. (f) Patient 6. (g) Legend.

**Figure 5.**
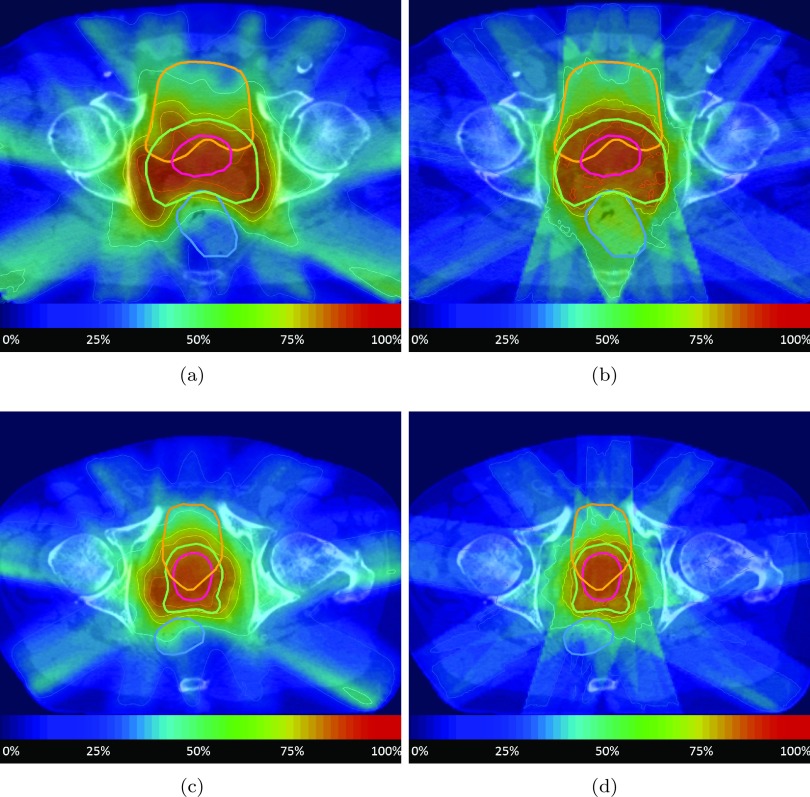
Dose distributions from patients 1 and 2. The color scale represents the dose value relative to the prescribed dose for the boost volume. Isodose lines are shown for 25, 50, 75, 85, 95 and 105% of the boost volume’s prescription. (a) Patient 1 IDS. (b) Patient 1 reference. (c) Patient 2 IDS. (d) Patient 2 reference.

The DVHs for each patient are shown in figure 4. The DVHs for the boost volume of the reference plan and the IDS generated plan agree fairly well for all 6 patients. However, the IDS plans generally showed a higher PTV dose than the reference plan which is particularly pronounced for patient 3. Despite the higher dose in the PTV, the rectum volume exposed to 25–45 Gy could be usually decreased, as it is clearly visible for patients 1–3, while the volume exposed to doses above 50 Gy did not increase significantly. For the bladder (indicated by the orange curves), IDS resulted in a somewhat larger volume for the dose range from 25 to 45 Gy for patient 1–3. The IDS plan had slightly less high dose (more than 70 Gy) for patient 5–6, slightly more high dose for patient 3–4, and similar high dose for patient 1–2. The body contour (purple curves) received a higher integral dose for all IDS plans. Representative dose distributions on a transversal CT slice for two patients are shown in figure 5.

The planning time per patient ranged from 15 to 45 minutes. The number of segments generated by the sequencer are listed in table 5 for both the IDS and reference plans. The reference plans all had a smaller amount of segments than the IDS plans. The number of segments ranged from 68 to 87 for the reference plans and from 84 to 99 for the IDS plans.

**Table 5.** Number of segments for the IDS and reference plans.

Patient	Segments IDS	Segments reference
Patient 1	94	87
Patient 2	87	68
Patient 3	84	81
Patient 4	95	76
Patient 5	96	79
Patient 6	99	86
Mean	92.5	79.5

## 4. Discussion

### 4.1. Treatment plan comparison

The quality of the IDS plans was comparable to the reference plans, in terms of both target coverage and OAR sparing. For some patients, the IDS algorithms had difficulties in separating the dose prescriptions for the PTV and boost volume, which resulted in non-separated DVH curves. A point of concern is the difference in integral dose, which is consistently higher for the IDS plans. On average, the sequenced IDS treatment plans consisted of 13 more segments than the reference plans. However, sequencing was performed with standard settings and an optimization of the number of segments was considered out of scope of this study.

Conventional IMRT optimization alike, the DMR strategy operates in the fluence domain. However, before the IDS treatment plan can be delivered, it has to be sequenced first. In practice, this might deteriorate the local dose manipulations and, thereby, the quality of the plan. Currently, we use an external sequencing algorithm, which is separated from the IDS algorithms. In the future, the DMR operation should be extended with an algorithm, which frequently checks whether the currently shaped dose distribution is still deliverable.

Treatment plan evaluation and comparison are non-trivial tasks. Thus, showing the potential of a new TPS, is not straightforward. A proof of concept study can only show the potential improvements. User acceptance, planning time, plan quality and plan approval can only be assessed in a large planning study.

### 4.2. Potential improvements

The IDS shortcomings, as identified in the previous section, are likely related to our strategy of dose recovery.

Spilling dose adjacent to the target volume (i.e. the second term of the *CI*) could be addressed by including the respective voxels in the DMR operation. However, the problem could also be reduced prior to dose shaping, by utilizing an alternative initial fluence distribution. The utilized algorithm takes only ${{D}_{50\%}}$ into consideration and does not try to achieve a steep dose gradient around the target volume. Alternatively, an initial dose distribution could potentially be derived from a conventionally optimized treatment plan, e.g. by use of a class solution.

The IDS plans all had a higher *NTID* than the reference plans, which clearly is a limitation of the current method. The occurrence of such relatively large low-dose baths potentially is a problem, as increasing evidence shows that these have a biological effect which has been underestimated in the past (Joiner and van der Kogel 2009). Part of the problem could be solved by reducing the dose spilled around the target volume. Therefore, the recovery algorithm has to be improved such that it takes into account voxels outside of the target volume, which requires an increase of the number of recovery steps *N*_*R*_ per DMR operation.

The body dose could also be reduced by user interaction with one of the IDS tools. Unfortunately, these localities typically do not have steep dose gradients and, hence, are not easily selectable. Moreover, a part of the body dose is caused solely by scattered radiation. In this case, the determination of a fluence patch is difficult.

The geometry-based voxel selection for the recovery algorithm works well for patient geometries with a few structures, as was the case for the prostate patients in this study. Future work incorporates the improvement of the dose shaping strategies for more complex patient geometries. Also, strategies will be implemented which allow for modification of voxels, based on inter-fractional changes in patient geometry.

The suggested improvements rely on a considerable increase of the amount of voxels to recover. As the number of recovered voxels *N*_*R*_ is proportional to the runtime of each DMR operation, this requires an ample speed-up of the dose calculation algorithm. Alternatively, an algorithm could be considered, which performs recovery as background process.

Although our IDS software allows for treatment planning from scratch, the initial phase of the planning process could be automatized aiming to generate good quality treatment plans more rapidly. The IDS tools could be used to tweak automatically optimized plans which are either generated on the fly or selected from a database. For automatic plan generation, either conventional IMRT planning methods could be exploited or an automated planning algorithm based on DMR could be investigated. In addition, the 3D graphical work-flow allows for the development of an algorithm for suggesting a set of dose modifications which indicate potential degrees of freedom directly in the dose visualization.

### 4.3. Related work

Other groups also make efforts to deal with the disadvantages of conventional IMRT treatment plan optimization as stated in the introduction. One example is automatic planning using a heuristic search based on constraint wish lists (Voet *et al* 2012) or plan databases (Wu *et al* 2011). Multi-criteria optimization (MCO) (Thieke *et al* 2007) makes the exploration of the penalty space feasible, but still suffers from the inability to quickly adapt a plan based on an updated patient geometry. Süss *et al* (2013) have extended MCO such that local cold or hot spots can be controlled indirectly. These methods are all computationally intensive and require many conventional optimization loops, which hampers the development of interactive and intuitive treatment planning tools. Therefore research is done on ultra-fast conventional IMRT plan optimization using various parallelization strategies (Men *et al* 2009, Ziegenhein *et al* 2013a). The IDS algorithm has the potential to cope with all before-mentioned drawbacks.

Recently, a similar planning approach was presented which allows for real-time interactive treatment planning as well (Otto 2014). Here, a software tool is utilized as pre-optimization step to facilitate dose shaping using a method similar to our DMR method. The user input is then converted to dose-volume constraints, which can be imported in a conventional TPS. Our IDS software, however, provides a fully interactive treatment planning environment, which does not require a conventional TPS and allows for 2D and 3D graphical user interaction. Our IDS software could be utilized in a similar indirect way to Otto (2014) and thereby support VMAT delivery techniques.

### 4.4. Summary and conclusion

We have shown a proof of concept study for IDS by comparing IMRT treatment plans for six prostate patients with reference plans. For this small cohort of patients we observed that a similar plan quality could be achieved for most clinical quality indicators. However, we also have observed that other relevant features, like the average integral dose, still need to be improved. Naturally, the DMR algorithms have to be evaluated for other, more challenging treatment sites in the future.

In conclusion, this work has shown that real-time treatment planning utilizing IDS is technically feasible for a first limited set of prostate patients. The observed limitations of our current implementation of the IDS strategy need to be addressed before an extended range of clinical applications should be considered.
